# Is There a Relationship Between the Histopathological Features and Anatomical Locations of Thrombi Obtained During Endovascular Thrombectomy in Acute Ischemic Stroke and Its Comorbid Diseases?

**DOI:** 10.3390/diagnostics16010063

**Published:** 2025-12-24

**Authors:** Saim Türkoğlu, Hüseyin Akdeniz, Ertuğ Günsoy, Fatma Ayaz Yalınkılıç

**Affiliations:** 1 Department of Radiology, Medical Faculty, Yuzuncu Yıl University, 65080 Van, Turkey; hakdenizdr@hotmail.com; 2 Emergency Clinic, Van Training and Research Hospital, University of Health Sciences, 65080 Van, Turkey; gunsoyertug@gmail.com; 3 Department of Pathology, Medical Faculty, Yuzuncu Yıl University, 65080 Van, Turkey; dr.fatma_ayaz@hotmail.com

**Keywords:** acute ischemic stroke, comorbidity, endovascular thrombectomy, histopathological, thrombi

## Abstract

**Objective:** This study aimed to assess the association between the histopathological characteristics of thrombi extracted during endovascular thrombectomy and clinical factors, including the location of the occlusion, comorbid conditions, and treatment effectiveness, in patients with acute ischemic stroke. **Materials and Methods:** A total of 57 patients with acute ischemic stroke who underwent endovascular thrombectomy between 1 January 2022 and 31 December 2024 were included in the study. Thrombi were analyzed histopathologically and classified into categories based on their composition (RBC-dominant, fibrin-dominant, RBC = fibrin, organized fibrin) and phase (early or late stage). CD34 staining was used to assess organized fibrin. **Results:** The mean age of the patients was 65.2 ± 15.3 years, 52.6% were female, and 47.4% were male. The majority of thrombi were retrieved from the MCA M1 segment (64.9%). Histopathological analysis revealed that 49.1% of thrombi were RBC-dominant, 21.1% RBC = fibrin, 19.3% fibrin-dominant, and 10.5% contained organized fibrin. Early-stage thrombi accounted for 70.2% of cases, while late-stage thrombi comprised 29.8%. Thrombus composition was significantly associated with anatomical location, with RBC-dominant thrombi being most prevalent in the proximal ICA (88.2%; *p* < 0.001). CD34 staining identified organized fibrin in 10.5% of thrombi, exclusively in patients who underwent stent placement. However, no statistically significant correlation was identified between CD34 positivity and thrombus composition (*p* > 0.05). Additionally, no notable associations were found between thrombus composition and chronic comorbidities. **Conclusions:** Thrombus composition and stage exhibit variability depending on anatomical location, particularly in the proximal ICA, where RBC-dominant thrombi are more frequent. Although CD34 positivity indicates organized fibrin, it does not show a significant relationship with thrombus characteristics or patient comorbidities. These findings underscore the complex interplay between thrombus histopathology, anatomical location, and procedural outcomes, highlighting the need for further investigation.

## 1. Introduction

The most effective strategy for preserving viable ischemic brain tissue in the treatment of acute ischemic stroke is the timely restoration of cerebral blood flow through reperfusion therapy [1]. In recent years, advanced treatments such as mechanical thrombectomy (MT) using catheter-based devices have become standard practice. MT has been demonstrated to significantly enhance outcomes in patients with acute large vessel occlusion stroke. Additionally, it offers a valuable opportunity for direct thrombus analysis in cases of cerebral embolism [2,3,4]. Investigating thrombi may provide valuable insights into the underlying mechanisms of stroke [3].

Previous studies have shown that thrombus formation occurs in distinct histological phases over time. Recent studies on the quantification of thrombus composition in ischemic stroke patients have primarily relied on conventional methods, including hematoxylin and eosin (H&E) staining. In addition to this, endothelial cell proliferation, detectable through CD34 immunostaining, serves as a surrogate marker for organized fibrin. This method aids in identifying thrombi that may be resistant in the later stages of their formation [3,4]. Thrombus composition is clinically important because RBC-rich clots tend to be softer and more amenable to aspiration or stent-retriever engagement, whereas fibrin-rich thrombi are mechanically stiffer, often requiring more device passes and being associated with lower reperfusion rates.

This study examined thrombus histopathology, analyzing its relationship with thrombus localization, patient comorbidities, and endovascular procedure success.

We hypothesized that distinct thrombus compositions—particularly the contrast between RBC-dominant and fibrin-dominant structures—exhibit different mechanical behaviors during thrombectomy and are associated with variation in final reperfusion outcomes

## 2. Materials and Methods

### 2.1. Trial Design

This study was conducted in the Radiology Department at the University of Van Yuzuncu Yil, Health Science, as a retrospective study. Ethical approval was obtained from the Ethics Committee of the University of Van Yuzuncu Yil, Health Science (Decision Number: GOKAEK/2025-01-16, Date: 24 January 2025). All participants provided written informed consent. The procedures involving human subjects were conducted in accordance with the 1964 Declaration of Helsinki.

### 2.2. Participants and Eligibility Criteria

The study included patients who presented with acute ischemic stroke and underwent endovascular thrombectomy between 1 January 2022 and 31 December 2024.

Inclusion criteria: acute ischemic stroke; for the anterior circulation system: patients with major vessel (ICA, ACA, MCA M1 and M2) occlusion with or without used tPA for the first 6 h or diffusion perfusion mismatch; for the posterior circulation system: within the first 24 h in patients with major vessel (basilar artery and PCA P1) occlusion; those undergoing endovascular thrombectomy; male or female; age > 18 years.

Exclusion criteria: for the anterior circulation system: patients with no diffusion perfusion mismatch, patients with suspected bleeding on non-contrast CT, an Alberta Stroke Program Early CT Score (ASPECTS) of less than 6, patients at risk of reperfusion bleeding, and patients undergoing other treatment modalities for acute ischemic stroke.

### 2.3. Parameters Evaluated

Demographic data, including age, gender, Modified Rankin Scale (mRS) scores, comorbidities, and the National Institutes of Health Stroke Scale (NIHSS) scores, were documented for all patients. In addition, the localizations where the thrombi caused occlusion, the modified TICI (mTICI) revascularization scale scores of the patients, and the histopathological content of the thrombi were examined and recorded. Some patients had more than one occlusion site, and more than one thrombus was removed. Thrombi were subdivided into early and late thrombi according to their content and into RBC-dominant, RBC and fibrin content equal, fibrin-dominant, and organized fibrin subgroups. These histopathological subgroups were compared according to occlusion localization, comorbidities, and mTICI scores, after which the study concluded.

### 2.4. Imaging Protocol

All participants underwent MRI prior to angiography unless contraindicated. Ischemic infarction was confirmed using diffusion-weighted imaging (DWI) for all individuals. MRI scans were performed on a 1.5-T scanner (Magnetom Symphony, Syngo MR B17; Siemens, Germany, 2009).

**Figure 1:**
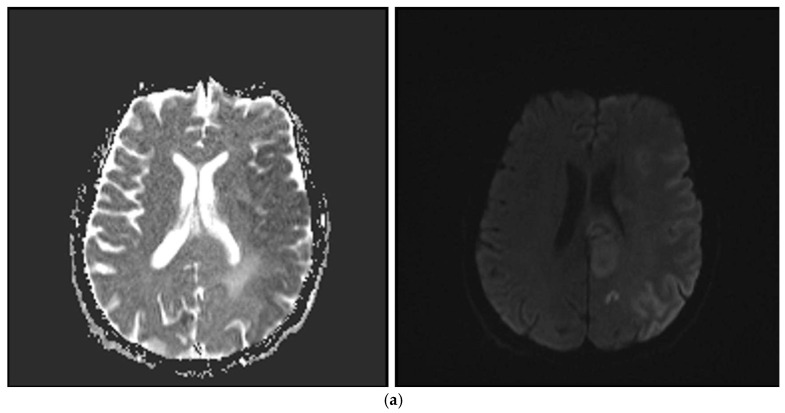

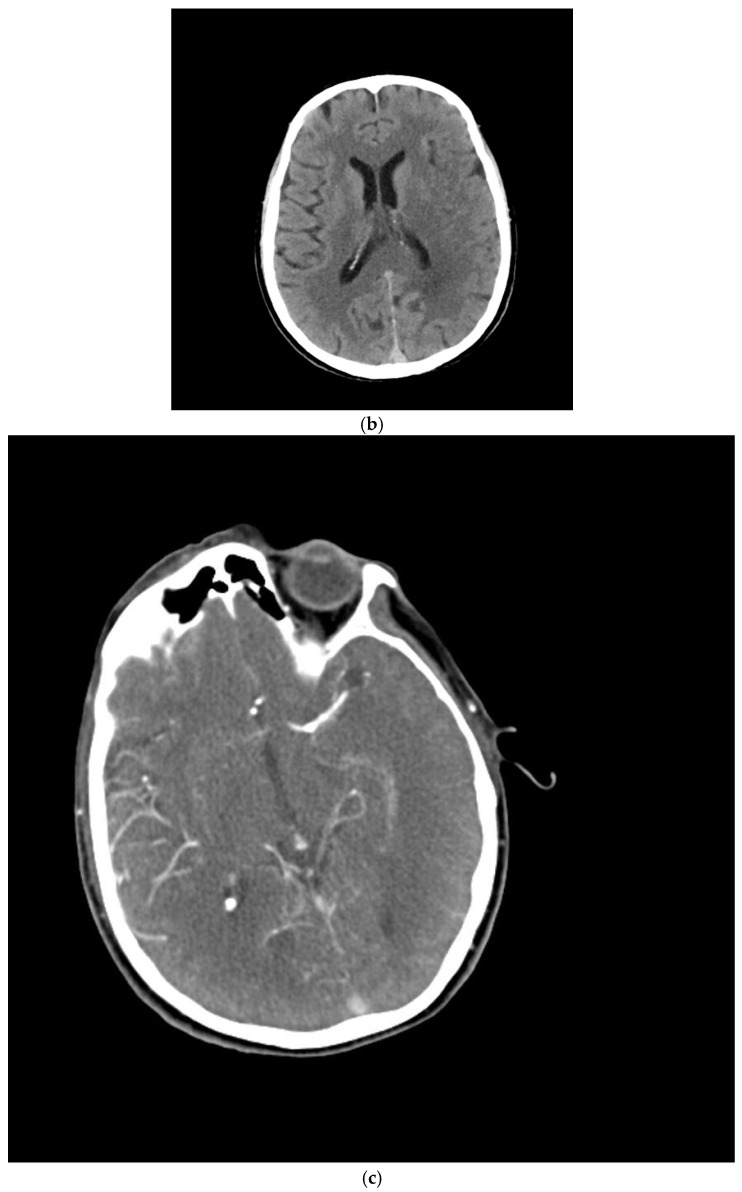
(**a**) ©DWI in a 73-year-old male patient; hypointense acute diffusion limitation in ADC affecting most of the left cerebral hemisphere; (**b**) faint hypodense area in the left cerebral hemisphere on non-contrast brain CT; (**c**) contrast-enhanced brain CT angiography shows left MCA M1 occluded from distal onwards.

All participants also underwent a non-contrast CT (NECT) scan with settings of 120 kV, 200 mAs, and a reconstructed slice thickness of 3 mm. Additionally, most patients received a multimodal CT exam, including CT angiography and CT perfusion, with parameters set to 80 kV, 100 mAs, and a reconstructed slice thickness of 1 mm. The thrombus location was identified through CT angiography or digital subtraction angiography (Figure 1a–c).

### 2.5. Endovascular Thrombectomy Technique

Endovascular thrombectomy (ET) was carried out using an aspiration catheter and/or a stent retriever under sedoanalgesia with a biplanar digital subtraction angiography (DSA) system (Artis_zee Biplane XA, Siemens, Munich, Germany). A guiding catheter was introduced transfemorally via the Seldinger technique and advanced toward the proximal segment of the suspected occlusion site.

Once the arterial occlusion was identified, a distal access or aspiration catheter was coaxially advanced over a microwire and, when feasible, positioned at the thrombus site. Thrombus aspiration was then performed using a VacLok syringe (AYSET, Seyhan, Adana, Türkiye). If recanalization remained insufficient, the aspiration process was repeated. Alternatively, a stent retriever was deployed, engaged with the thrombus, and one or more retrieval attempts were conducted under continuous aspiration.

**Figure 2:**
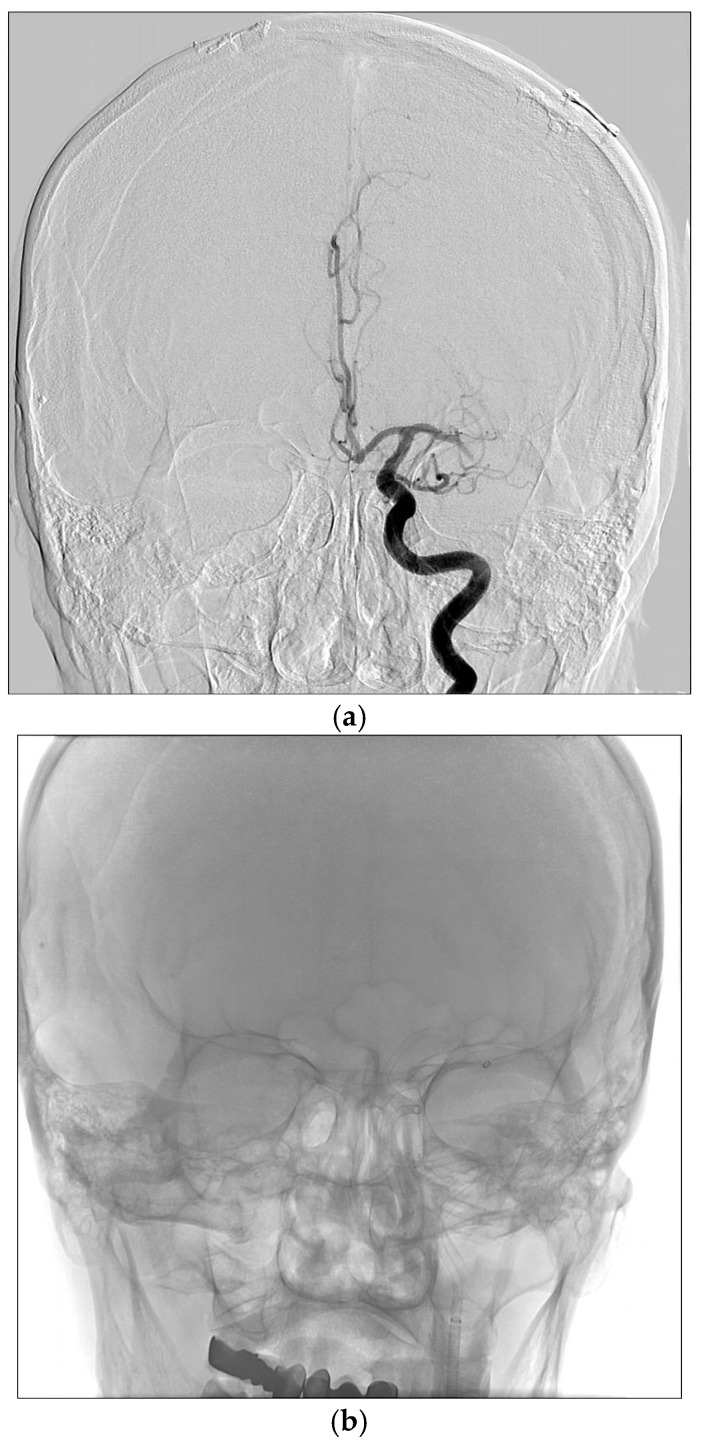

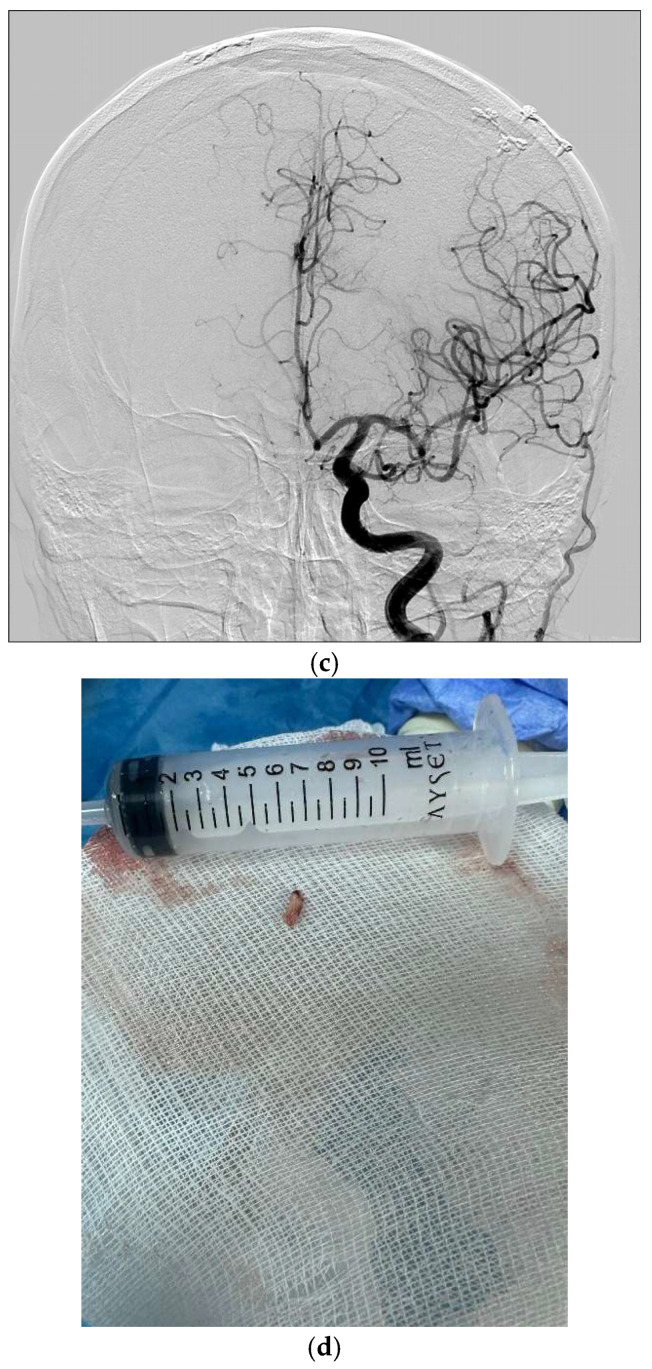
(**a**) Digital subtraction angiography performed 4 h after symptom onset; occlusion in the left MCA M1; (**b**) scopy image of the patient who was aspirated for 2 min by advancing a 6F neurocatch catheter to distal left MCA M1 for thrombectomy; (**c**) a patient with total patency after thrombectomy m(TICI 3); (**d**) clot material removed at thrombectomy (histopathological diagnosis of organized fibrin).

Thrombi were collected under sterile conditions, and those deemed suitable for histopathological examination were immediately preserved in formalin (Figure 2a–d).

### 2.6. Histopathological Examination

Thrombectomy materials were stained with hematoxylin and eosin (H&E), and CD34 immunostaining was performed to identify endothelial cells. CD34 positivity was evaluated because endothelial cells and endothelial-derived fibroblastic elements may become incorporated into late-stage, organized fibrin; therefore, CD34 staining can indicate endothelial integration within an organized thrombus

A pathologist, blinded to the study details, conducted the histological analysis of the thrombus samples. The thrombi were classified into four categories: RBC-dominant, a balanced mixture of RBCs and fibrin, fibrin-dominant, and organized fibrin. Fibrin organization was evaluated based on endothelial cell ingrowth, as seen in CD34-immunostained sections. Additionally, thrombus pathology was divided into early-phase (RBC-dominant or RBC-fibrin balanced) and late-phase (fibrin-dominant or organized fibrin) groups.

### 2.7. Statistical Analysis

Statistical analysis was conducted using Jamovi software (version 2.6.17.0). Continuous variables with normal distribution are expressed as mean ± standard deviation with 95% confidence intervals. For non-normally distributed variables, data are presented as medians with interquartile ranges (IQR: 25th–75th percentiles). Fisher’s exact test was applied to compare categorical variables, with a *p*-value of <0.05 indicating statistical significance.

## 3. Results

This study involved 57 patients diagnosed with acute ischemic stroke who underwent endovascular thrombectomy between 1 January 2022 and 31 December 2024. The average age of participants was 65.2 ± 15.3 years, with 52.6% being female. Chronic comorbidities were present in 63.1% of patients, with the most common being hypertension (31.6%), followed by coronary artery disease (24.6%), diabetes mellitus (19.3%), atrial fibrillation (12.3%), and a history of prior stroke (10.5%) (Table 1).

The mean NIHSS score was 11.1 ± 4.7, and the median mRS score was 4 (IQR: 2–5). Thrombi were extracted from different arterial locations, with the majority located in the M1 segment of the middle cerebral artery (MCA) (*n* = 37, 64.9%). Other thrombus locations included the proximal internal carotid artery (ICA) (*n* = 17, 29.8%), its terminal bifurcation (*n* = 11, 19.3%), the anterior cerebral artery (ACA) (*n* = 5, 8.8%), and the basilar artery (*n* = 2, 3.5%) (Table 2).

**Figure 3:**
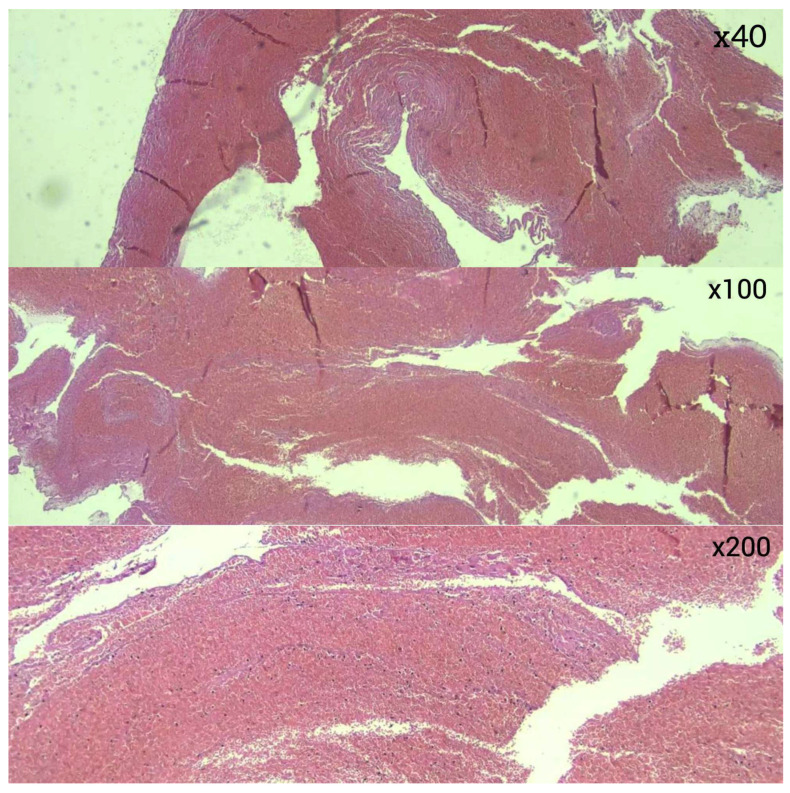
RBC-dominant thrombus (hematoxylin–eosin).

**Figure 4:**
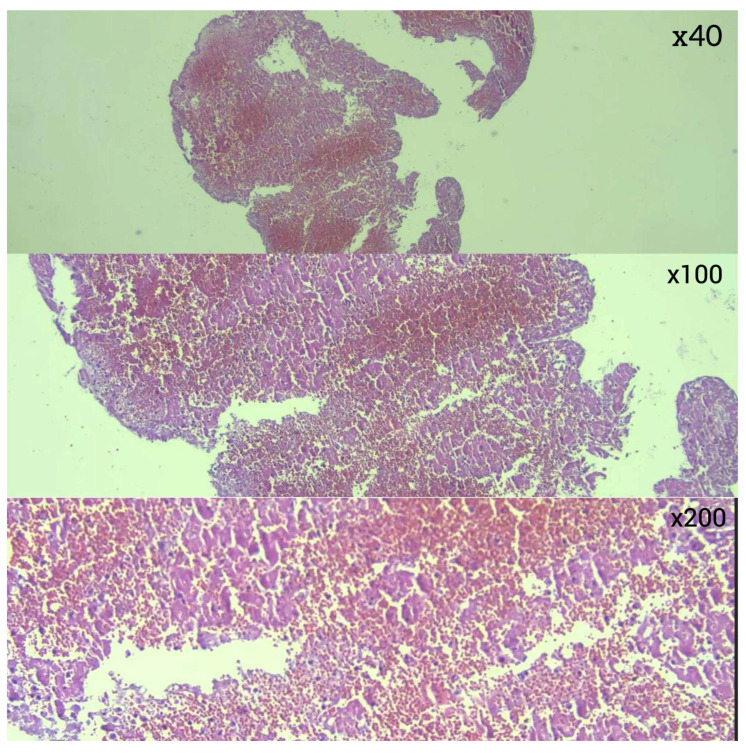
Thrombus with equal red blood cell and fibrin content (hematoxylin–eosin).

**Figure 5:**
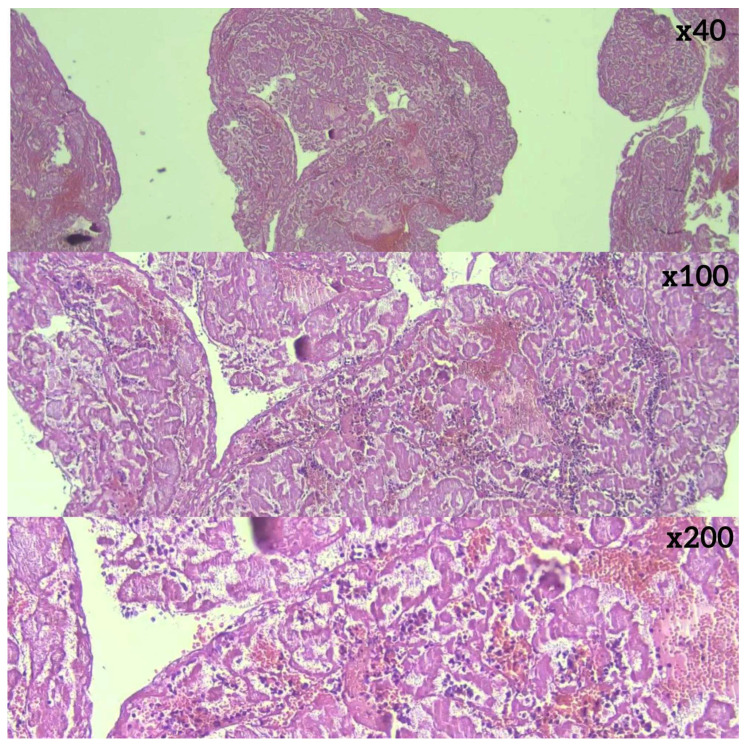
Fibrin-dominant thrombus (hematoxylin–eosin).

**Figure 6:**
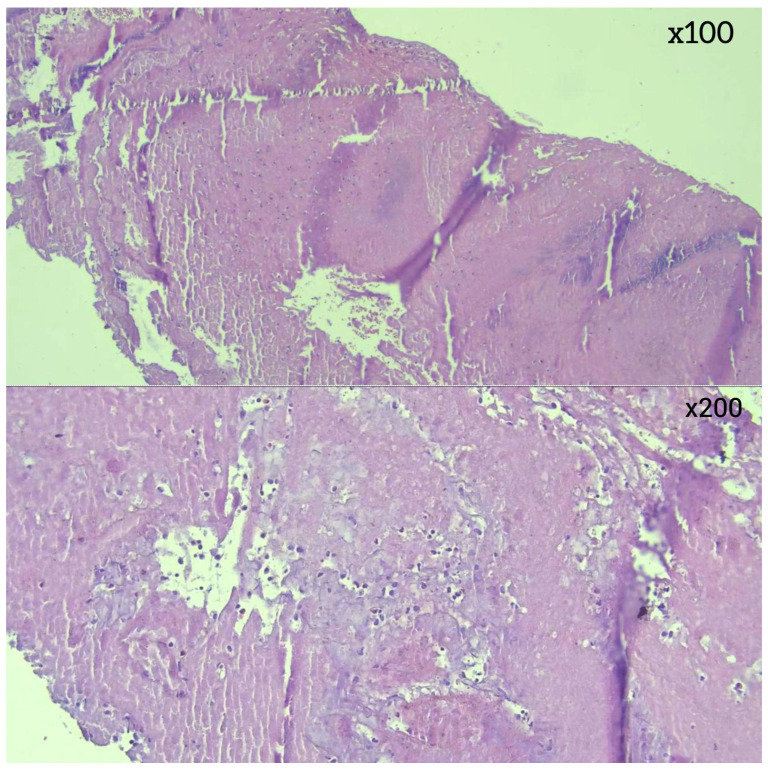
Organized fibrin (hematoxylin–eosin).

Histopathological analysis revealed differences in thrombus composition. RBC-dominant thrombi were the most common, observed in 28 (49.1%) of cases. (Figure 3) This was followed by thrombi with equal proportions of red blood cells and fibrin (*n* = 12, 21.1%) (Figure 4), fibrin-dominant thrombi (*n* = 11, 19.3%) (Figure 5), and thrombi with organized fibrin (*n* = 6, 10.5%) (Figure 6). In terms of thrombus phases, 40 cases (70.2%) were classified as early-stage, while 17 (29.8%) were classified as late-stage (Table 2 and Table 3). RBC and fibrin values were analyzed semi-quantitatively by measuring the areas covered as a percentage.

RBC-dominant thrombi were identified in several anatomical locations but were particularly prevalent in the ICA proximal segment, where 15 (88.2%) of thrombi were RBC-dominant (*p* < 0.001). In contrast, fibrin-dominant thrombi were less frequent overall but showed variability across locations. For example, fibrin-dominant thrombi were present in the MCA M1 region (*n* = 8, 21.6%) and ICA terminal bifurcation (*n* = 2, 18.2%) without reaching statistical significance in these sites (*p* > 0.05). Thrombi composition in other locations, such as the ACA and basilar artery, did not show significant associations with histopathological composition due to the limited sample size.

There was a significant difference in thrombus composition across occlusion sites, driven by the predominance of RBC-dominant thrombi in the proximal ICA and MCA M1 segments, and the clustering of fibrin-dominant and organized fibrin thrombi in the MCA M1 and ICA terminal locations (*p* < 0.001) (Table 3).

No significant relationship was observed between comorbidities and thrombus histopathological characteristics (*p* > 0.05) (Table 4 and Table 5).

Of the patients who underwent embolectomy, 35 (61.5%) underwent stent placement after aspiration, while 22 (39.6%) underwent aspiration alone. When the groups were compared based on thrombus subtypes, aspiration followed by stent placement was the most common procedure across all groups. Additionally, both aspiration and post-aspiration stenting procedures were most frequently performed in patients with RBC-dominant thrombi (*p* = 0.54). Similarly, both procedures were most commonly performed in early-stage thrombi (*p* = 0.35) (Table 2, Table 3 and Table 4).

Organized fibrin was detected in 10.5% of all thrombi. The distribution of the CD 34 positivity was assessed based on thrombus composition and stage, but no statistically significant associations were observed between CD 34 staining and thrombus histopathological categories (*p* > 0.05) (Table 4 and Table 5).

**Figure 7:**
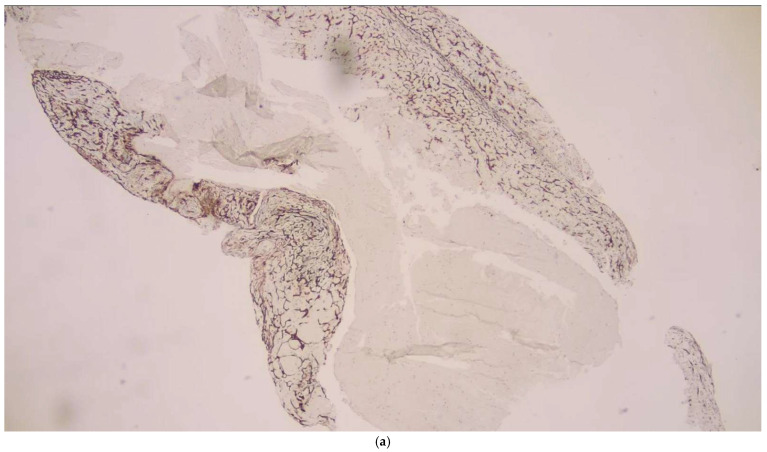

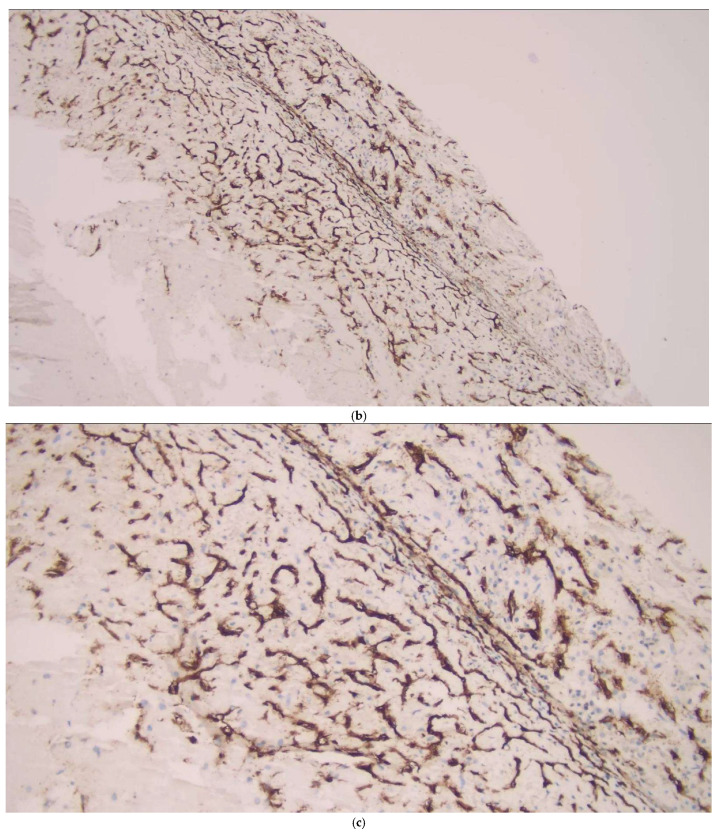
(**a**) CD34-positive staining in endothelial cells in organized fibrin-IHK ×40; (**b**) CD34-positive staining in endothelial cells in organized fibrin-IHK ×100; (**c**) CD34-positive staining in endothelial cells in organized fibrin-IHK ×200.

**Figure 8:**
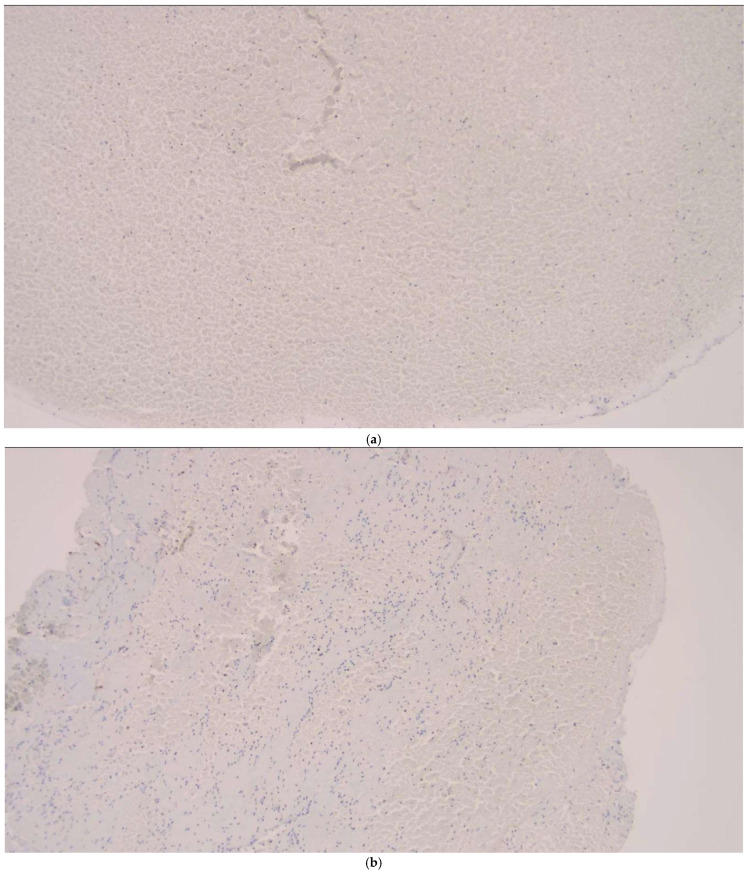

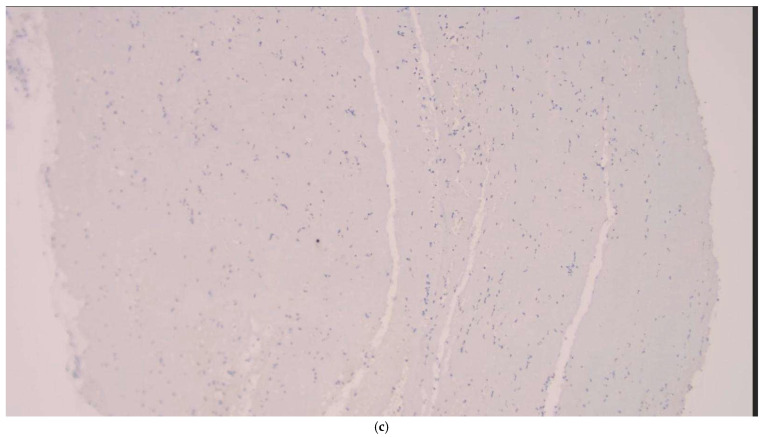
(**a**) CD34 negativity in RBC-dominant thrombus tissue-IHC ×100; (**b**) CD34 negativity in RBC and fibrin equal thrombus tissue-IHC ×100; (**c**) CD34 negativity in fibrin-dominant tissue-IHC ×100.

In the analysis of thrombus phases, CD34 positivity was observed slightly more frequently in late-stage thrombi (11.8%) compared to early-stage thrombi (7.5%), though this difference was not statistically significant (*p* = 0.629) (Figure 7). In terms of thrombus composition, organized fibrin was detected exclusively within a subset of fibrin-dominant thrombi and those with an equal proportion of RBCs and fibrin. However, the variation in CD34 positivity across these categories did not reach statistical significance (*p* = 0.167) (Figure 8).

Among the patients who achieved complete reperfusion (mTICI 3), 55% had RBC-dominant thrombi, making this the most effective thrombus type for achieving complete reperfusion. Additionally, 20% of thrombi in this group were RBC = fibrin, 17.5% were fibrin-dominant, and 7.5% contained organized fibrin. Notably, 78.6% of all RBC-dominant thrombi were associated with complete reperfusion. (Table 4 and Table 5). A dedicated comparison of CD34-positive and CD34-negative thrombi showed no significant differences in thrombus composition, occlusion site, or reperfusion outcomes.

The distribution of thrombus subtypes across TOAST etiologies is presented in Table 6. In large-artery atherosclerosis, RBC-dominant thrombi constituted the highest proportion (47%), whereas fibrin-dominant thrombi were relatively more frequent in cardioembolic strokes (31%). No TOAST 3 cases were observed, as small-vessel occlusions are not treated with mechanical thrombectomy. Final reperfusion outcomes differed across TOAST categories (Table 7), with mTICI 3 rates of 71% in TOAST 1, 77% in TOAST 2, 100% in TOAST 4, and 59% in TOAST 5, and these patterns were consistent with the higher procedural success typically observed in cases dominated by RBC-rich thrombi.

In patients with partial reperfusion (mTICI 2), RBC-dominant thrombi were still the most frequent, accounting for 35.7% of cases. RBC = fibrin thrombi represented 28.6%, fibrin-dominant thrombi 14.3%, and organized fibrin 21.4%. Organized fibrin was more prevalent in this group (50%). In cases of minimal reperfusion (mTICI 1), RBC-dominant and fibrin-dominant thrombi were evenly distributed, each accounting for 50% of cases. RBC = fibrin thrombi and organized fibrin were not observed in this group. In the no-reperfusion group (mTICI 0), all thrombi were fibrin-dominant (100%), highlighting the challenging nature of this thrombus type for achieving reperfusion. The statistical analysis using Fisher’s exact test (*p* = 0.393) showed no statistically significant association between thrombus histopathology and mTICI scores (Table 4 and Table 5).

## 4. Discussion

The histopathological characteristics of thrombi in acute ischemic stroke remain underexplored. Existing studies are limited, often report highly variable results, and typically involve small sample sizes. We believe this study makes a significant contribution to the literature in terms of both sample size and findings.

Boodt et al. and Shimizu et al. reported that thrombus was most frequently detected in the M1 segment of the MCA [1,2]. Our study confirmed the M1 segment of the MCA as the most frequent occlusion site, followed by the proximal ICA, ICA’s terminal bifurcation, ACA, and basilar artery.

When Mereuta et al. analyzed thrombi, they reported that RBC-dominant thrombi were the most common, followed by fibrin-dominant thrombi and thrombi composed of platelets and other blood cells [4]. Similarly, Shimizu et al. found that fibrin-dominant thrombi were the most common, followed by RBC-dominant and platelet-dominant thrombi [2]. In contrast, Schuhman et al. reported that these histopathological subtypes occurred with equal frequency [5]. In our study, RBC-dominant thrombi were the most common, followed by those with equal RBC and fibrin content, fibrin-dominant thrombi, and those with organized fibrin content. These findings highlight the variability in thrombus composition across studies. It is important to recognize that retrieved thrombi display considerable heterogeneity in both their composition and organization. As a result, the common components found within them may influence their response to treatment for acute ischemic stroke.

Early-stage thrombi are characterized by RBC predominance or an equal RBC-fibrin content, whereas late-stage thrombi exhibit fibrin predominance and organized fibrin content. In this regard, Simons et al. found that most patients had early-stage thrombi [3]. Conversely, Shimizu et al. reported that thrombi were late-stage in most patients [2]. In our study, the majority of thrombi were classified as early-stage. These differences are due to the different proportions of ingredients present in the thrombus.

Marder et al. reported that thrombi with mixed histopathology were most commonly localized in the ICA, fibrin-predominant thrombi in the ICA, erythrocyte-predominant thrombi in the MCA, and thrombi containing other blood elements in the MCA [6]. In the study by Shimizu et al., platelet-rich thrombi were most commonly localized in the MCA M1, RBC-dominant thrombi in the ICA and MCA M1, and VWF-dominant thrombi in both the ICA and MCA M1 [2]. In our study, all thrombus types except RBC-dominant thrombi were most commonly localized in the MCA M1. RBC-dominant thrombi were most frequently found in the proximal ICA and MCA M1 (*p* < 0.001). Our findings were consistent with other studies in the literature.

Several previous studies have investigated the relationship between stroke etiology and thrombus composition; however, no significant differences were found [2,6,7]. In general, acute ischemic stroke was primarily attributed to cardioembolic causes [6]. However, the relationship between comorbidities and thrombus composition has rarely been addressed in previous studies. In one of these, thrombi from patients with diabetes were studied, and it was found that clots in patients with diabetes had more fibrin and less erythrocyte components compared to those in patients without diabetes [8]. In our study, we examined this aspect and found that RBC-dominant thrombi were proportionally most common in almost all comorbidities, but no statistically significant association was observed between comorbidities and thrombus composition.

Traditionally, thrombus composition has been classified as “red thrombus,” “white thrombus,” or mixed. However, with the introduction of CD34 immunostaining for endothelial cell detection, a fourth category—organized fibrin—has been clearly defined. In a study utilizing CD34 immunostaining to assess organized fibrin pathology, 11 thrombus samples (27.5%) tested positive for endothelial cell growth [3,9,10,11]. In our study, CD34 positivity was observed in 2 cases within the organized fibrin category, supporting this classification. However, no significant differences were found among thrombus histopathological subtypes in terms of CD34 staining. These findings suggest that while CD34 staining provides insight into the presence of organized fibrin, its distribution is not significantly associated with thrombus composition or phase. This emphasizes the need for further studies to explore the implications of CD34 positivity in thrombus pathology and its potential relationship with clinical outcomes.

Studies have shown that vessels blocked by fibrin-dominant thrombi require more passes with a thrombectomy device to achieve recanalization than those blocked by RBC-rich thrombi [12,13,14]. The increased fibrin content makes the clot stiffer and more elastic, which reduces the likelihood of successful interaction with the thrombectomy device. The thrombus’s mechanical stability—characterized by its stiffness, elasticity, and rigidity—is largely influenced by its fibrin composition [15]. Furthermore, studies have shown that recanalization is typically achieved more rapidly in platelet-rich clots, leading to shorter recanalization times [7]. However, some research has found no significant correlation between thrombus characteristics and recanalization success or overall procedure duration [3]. In our study, we did not analyze data regarding total procedure time or the number of passes needed for recanalization. However, no substantial differences were noted between thrombus subtypes in terms of the proportion of patients who received stenting in addition to aspiration.

The extent of reperfusion following recanalization, assessed using the mTICI scale, serves as an important indicator of procedural success. Numerous studies have explored the link between thrombus composition and mTICI scores. While some found no significant correlation [16,17,18,19], others suggested that RBC-dominant thrombi were linked to more favorable recanalization outcomes (mTICI > 2b) compared to fibrin- and platelet-rich thrombi [20,21,22,23,24]. The relationship between mTICI scores and thrombus histopathology reveals valuable insights. The majority of patients who achieved complete reperfusion (mTICI 3) had RBC-dominant thrombi, making this the most effective thrombus type for achieving complete reperfusion. Furthermore, the other thrombus types in this group included RBC = fibrin, fibrin-dominant, and organized fibrin. Notably, 78.6% of all RBC-dominant thrombi were associated with complete reperfusion. This suggests that RBC-dominant thrombi yield better recanalization outcomes. In patients with partial reperfusion (mTICI 2), RBC-dominant thrombi were still the most frequent, accounting for 35.7% of cases. Furthermore, the other thrombus types in this group included RBC = fibrin, fibrin-dominant, and organized fibrin. Organized fibrin was more prevalent in this group (50%), suggesting that it may hinder complete reperfusion, though partial restoration of blood flow was achievable.

In cases of minimal reperfusion (mTICI 1), RBC-dominant and fibrin-dominant thrombi were evenly distributed, each accounting for 50% of cases. RBC = fibrin thrombi and organized fibrin were not observed in this group. These findings indicate that fibrin-dominant thrombi may be more resistant to mechanical thrombectomy, limiting reperfusion success.

In the no-reperfusion group (mTICI 0), all thrombi were fibrin-dominant (100%), highlighting the challenging nature of this thrombus type for achieving reperfusion. Fibrin-dominant thrombi are likely more resistant to removal, contributing to complete reperfusion failure.

The statistical analysis using Fisher’s exact test (*p* = 0.393) showed no statistically significant association between thrombus histopathology and mTICI scores. However, trends observed in the data suggest that thrombus composition plays a critical role in reperfusion success. RBC-dominant thrombi are more frequently associated with complete reperfusion and appear easier to retrieve during thrombectomy. In contrast, fibrin-dominant thrombi are more challenging, often leading to minimal or no reperfusion. Organized fibrin, while less common, is associated with partial reperfusion and may act as a barrier to achieving complete reperfusion. These results underscore the significance of thrombus histopathology in predicting the success of endovascular treatments and stress the importance of personalized therapeutic approaches.

This study had a number of limitations. One of them was its single-center retrospective design. Additionally, there were factors contributing to inherent selection bias. For instance, the administration of intravenous r-tPA might have influenced the sample characteristics. The thrombus material collected may not always have represented the entire thrombus. It is also possible that more stable clot components were more easily removed by thrombectomy. Only thrombi that were not successfully lysed or retrieved by thrombectomy were analyzed, which excluded those that could not be retrieved or were sensitive to thrombolysis. The generalizability of location-specific findings is limited by the very small number of posterior circulation occlusions (*n* = 2) in our cohort.

Detailed procedural metrics such as pass number, early re-occlusion, in situ thrombosis, or device-related difficulty were not recorded in our dataset, which prevented evaluation of potential associations with CD34 positivity.

Lastly, it should be acknowledged that some thrombi may have formed due to catheter manipulations, although this occurrence was rare.

In our study, the most common occlusion site was the M1 segment of the MCA. Most thrombi were early stage, with RBC-dominant thrombi being the most prevalent. RBC-dominant thrombi mainly involved the proximal ICA and MCA M1, while other types were predominantly in MCA M1. RBC-dominant thrombi showed better reperfusion outcomes, whereas fibrin-dominant ones were linked to worse outcomes. These findings highlight the role of thrombus histopathology in predicting endovascular treatment success and the need for individualized therapies.

## Figures and Tables

**Table 1:** Demographic characteristics of the patients.

Characteristic	*n* = 57
**Age**	65.2 (±15.3)
**Gender (Male/Female)**	27/30
**Chronic Diseases (CD)**	
Diabetes Mellitus	11 (19.3%)
Atrial Fibrillation	7 (12.3%)
Stroke	6 (10.5%)
Coronary Artery Disease	14 (24.6%)
Hypertension	18 (31.6%)
Any CD	36 (63.1%)

CD: chronic diseases.

**Table 2:** Clinical characteristics of the patients.

Characteristic	*n* = 57
**NIHSS scores**	11.1 (±4.7)
**mRS scores**	4 (2–5)
**Occlusion Location**	
MCA M1	37 (64.9%)
ICA terminal	11 (19.3%)
ICA proximal	17 (29.8%)
ACA	5 (8.8%)
Basilar artery	2 (3.5%)
**Embolectomy Technique**	
Aspiration	22 (39.6%)
Aspiration and stent implantation	35 (61.4%)

NIHSS: the National Institutes of Health Stroke Scale, mRS: Modified Rankin Scale, MCA: middle cerebral artery, ICA: internal carotid artery, ACA: anterior cerebral artery.

**Table 3:** Histopathological classification of thrombin.

Characteristic	*n* = 57	MCA M1	ICA Terminal	ICA Proximal	ACA	Basilar Artery	*p* Value
**Early Stage**	40 (70.2%)						
RBC-dominant	28 (49.1%)	14 (38.9%)	3 (8.3%)	15 (41.7%)	2 (5.5%)	2 (5.5%)	**<0.001**
RBC = Fibrin	12 (21.1%)	10 (66.7%)	4 (2.7% )	0	1 (6.7%)	0	**<0.001**
**Late Stage**	17 (29.8%)						
Fibrin-dominant	11 (19.3%)	8 (66.7%)	2 (16.7%)	1 (8.3%)	1 (8.3%)	0	**<0.001**
Organized fibrin	6 (10.5%)	5 (55.5%)	2 (22.2%)	1 (11.1%)	1 (11.1%)	0	**<0.001**
***p* Value**		0.15	0.25	**<0.001**	0.89	-	

MCA: middle cerebral artery, ICA: internal carotid artery, ACA: anterior cerebral artery.

**Table 4:** Comparison of clinical features according to thrombin classification.

	RBC-Dominant	RBC = Fibrin	Fibrin-Dominant	Organized Fibrin	*p* Value
**CD 34**	2 (7.1%)	1 (8.3%)	0	2 (33.3%)	0.17
**Chronic Diseases**					
Hypertension	9 (50%)	5 (27.8%)	3 (16.7%)	1 (5.6%)	0.82
Coronary Artery Disease	9 (64.3%)	3 (21.4%)	1 (7.1%)	1 (7.1%)	0.59
Diabetes Mellitus	6 (54.5%)	2 (18.2%)	3 (27.3%)	0	0.67
Atrial Fibrillation	2 (28.6%)	1 (14.3%)	2 (28.6%)	2 (28.6%)	0.22
Stroke	3 (50%)	0	2 (33%)	1 (16.7%)	0.39
**Embolectomy Technique**					0.54
Aspiration	12 (54.5%)	5 (22.7%)	5 (22.7%)	0	
Aspiration and stent implantation	16 (45.7%)	7 (20%)	6 (17.1%)	6 (17.1%)	
**mTICI Score**					0.39
3	22 (55%)	8 (20%)	7 (17.5%)	3 (7.5%)	
2	5 (35.7%)	4 (28.6%)	2 (14.3%)	3 (21.4%)	
1	1 (50%)	0	1 (50%)	0	
0	0	0	1 (100%)	0	

mTICI: modified TICI.

**Table 5:** Comparison of clinical features of early and late thrombin.

	Early	Late	*p* Value
**CD 34**	3 (7.5%)	2 (11.8%)	0.63
**Occlusion Location**			
MCA M1	24 (64.8%)	13 (35.2%)	0.23
ICA terminal	7 (63.7%)	4 (36.3%)	0.59
ICA proximal	15 (88.2%)	2 (11.8%)	0.052
ACA	3 (60%)	2 (40%)	0.63
Basilar artery	2 (100%)	0	-
**Chronic Diseases**			
Hypertension	14 (77.8%)	4 (22.2%)	0.54
Coronary Artery Disease	12 (85.7%)	2 (14.3%)	0.19
Diabetes Mellitus	8 (72.7%)	3 (27.3%)	0.84
Atrial Fibrillation	3 (42.9%)	4 (57.1%)	0.18
Stroke	3 (50%)	3 (50%)	0.35
**Embolectomy Technique**			0.35
Aspiration	17 (77.3%)	5 (22.7%)	
Aspiration and stent implantation	23 (65.7%)	12 (34.3%)	
**mTICI Score**			0.26
3	30 (75%)	10 (25%)	
2	9 (64.3%)	5 (25.7%)	
1	1 (50%)	1 (50%)	
0	0	1 (100%)	

MCA: middle cerebral artery, ICA: internal carotid artery, ACA: anterior cerebral artery, mTICI: modified TICI.

**Table 6:** Distribution of histopathological thrombus subtypes according to toast classification.

TOAST Etiology	RBC-Dominant	RBC = Fibrin	Fibrin-Dominant	Organized Fibrin	Total (*n*)
TOAST 1—Large artery atherosclerosis	8 (47%)	5 (29%)	3 (18%)	1 (6%)	17
TOAST 2—Cardioembolic	6 (46%)	1 (8%)	4 (31%)	2 (15%)	13
TOAST 4—Other determined	4 (80%)	1 (20%)	0 (0%)	0 (0%)	5
TOAST 5—Undetermined	10 (45%)	5 (23%)	4 (18%)	3 (14%)	22

**Table 7:** Final reperfusion grades (mTICI) across TOAST etiologies.

TOAST Etiology	mTICI 0	mTICI 1	mTICI 2	mTICI 3	Total (*n*)
TOAST 1—Large artery atherosclerosis	0 (0%)	1 (6%)	4 (24%)	12 (71%)	17
TOAST 2—Cardioembolic	0 (0%)	0 (0%)	3 (23%)	10 (77%)	13
TOAST 4—Other determined	0 (0%)	0 (0%)	0 (0%)	5 (100%)	5
TOAST 5—Undetermined	1 (5%)	1 (5%)	7 (32%)	13 (59%)	22

## Data Availability

The original contributions presented in this study are included in the article. Further inquiries can be directed to the corresponding author.
